# Supplementary material for: Combined Modeling Study of the Binding Characteristics of Natural Compounds, Derived from Psoralea Fruits, to β-Amyloid Peptide Monomer
Source: Int J Mol Sci. 2022 Mar 24;23(7):3546. doi: 10.3390/ijms23073546 (PMC8998326; doi:10.3390/ijms23073546)
Supplement: Supplementary file 1 [file ijms-23-03546-s001.zip › ijms-1590806-supplementary.pdf]

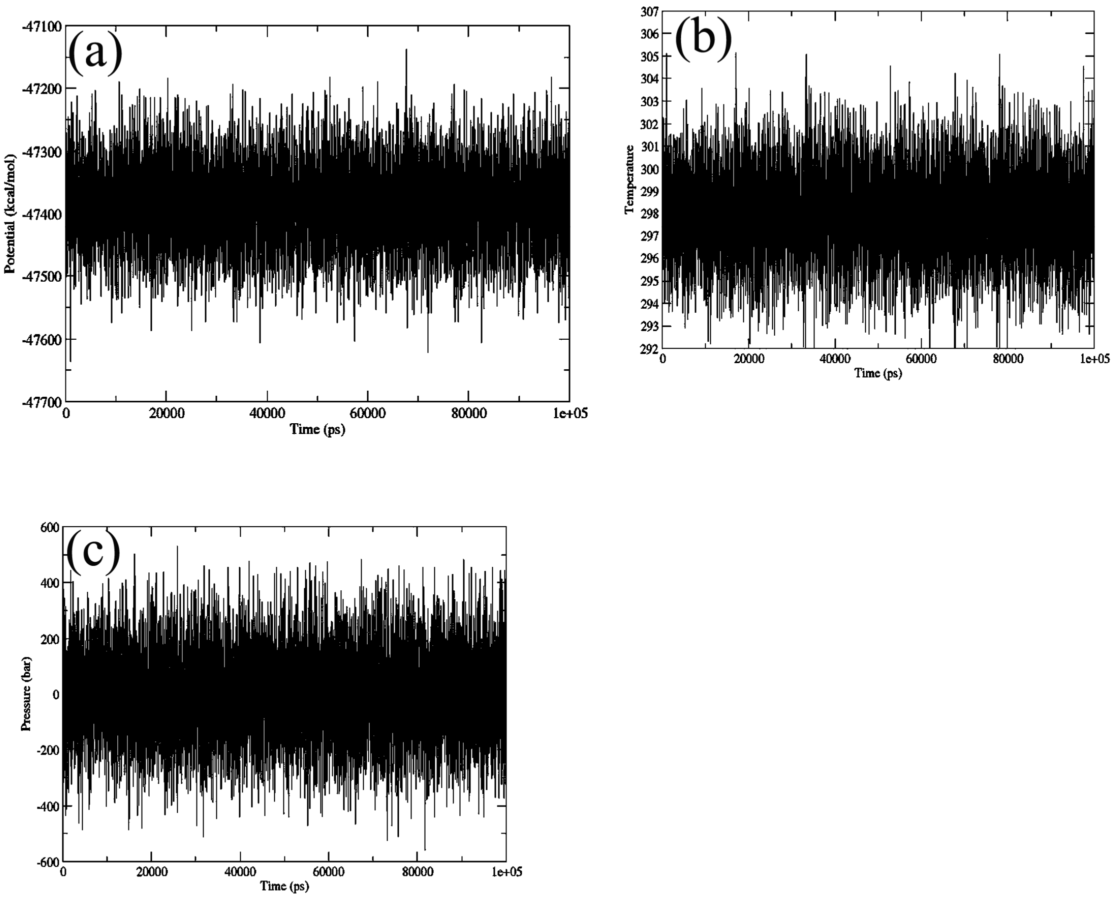

Figure S1. Compound 1 MD simulations system: Plot of the distribution of the potential energy (**a**), temperature (**b**) and pressure (**c**).

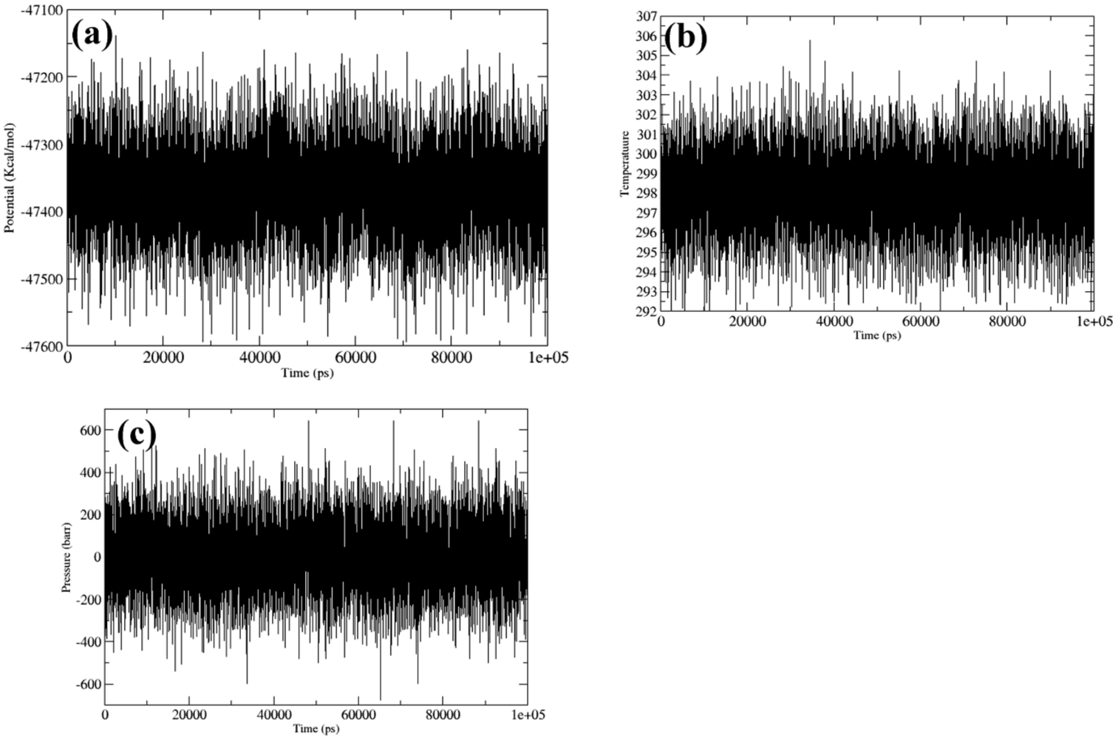

Figure S2. Compound 2 MD simulations system: Plot of the distribution of the potential energy (**a**), temperature (**b**) and pressure (**c**).

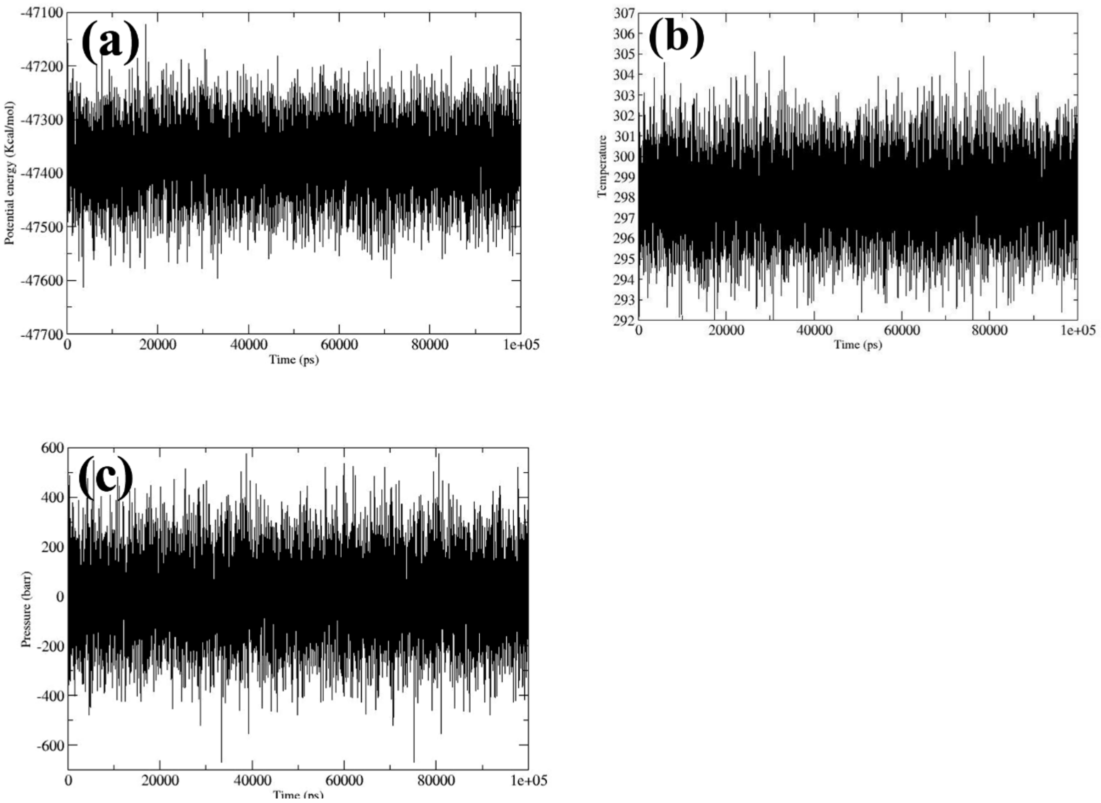

Figure S3. Compound 3 MD simulations system: Plot of the distribution of the potential energy (**a**), temperature (**b**) and pressure (**c**).

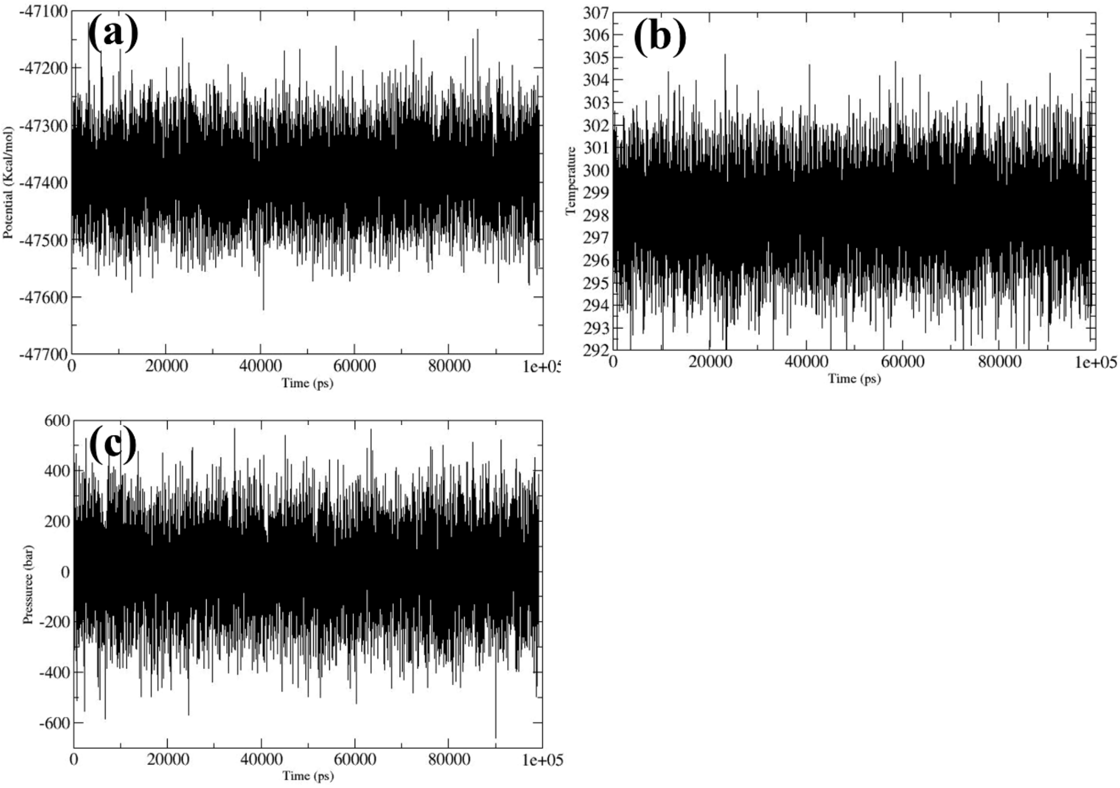

Figure S4. Compound 4 MD simulations system: Plot of the distribution of the potential energy (**a**), temperature (**b**) and pressure (**c**).

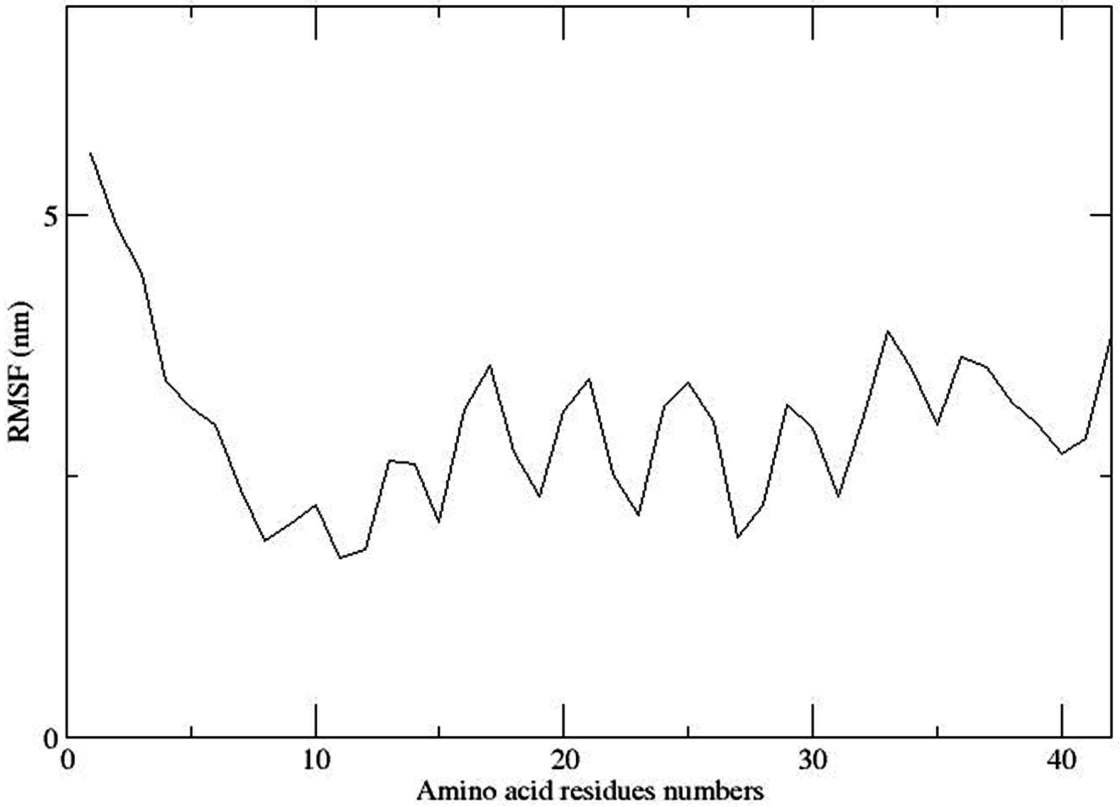

Figure S5. Root mean square fluctuation (RMSF) plot of protein residues of compound 1-AB42 MD simulations system.

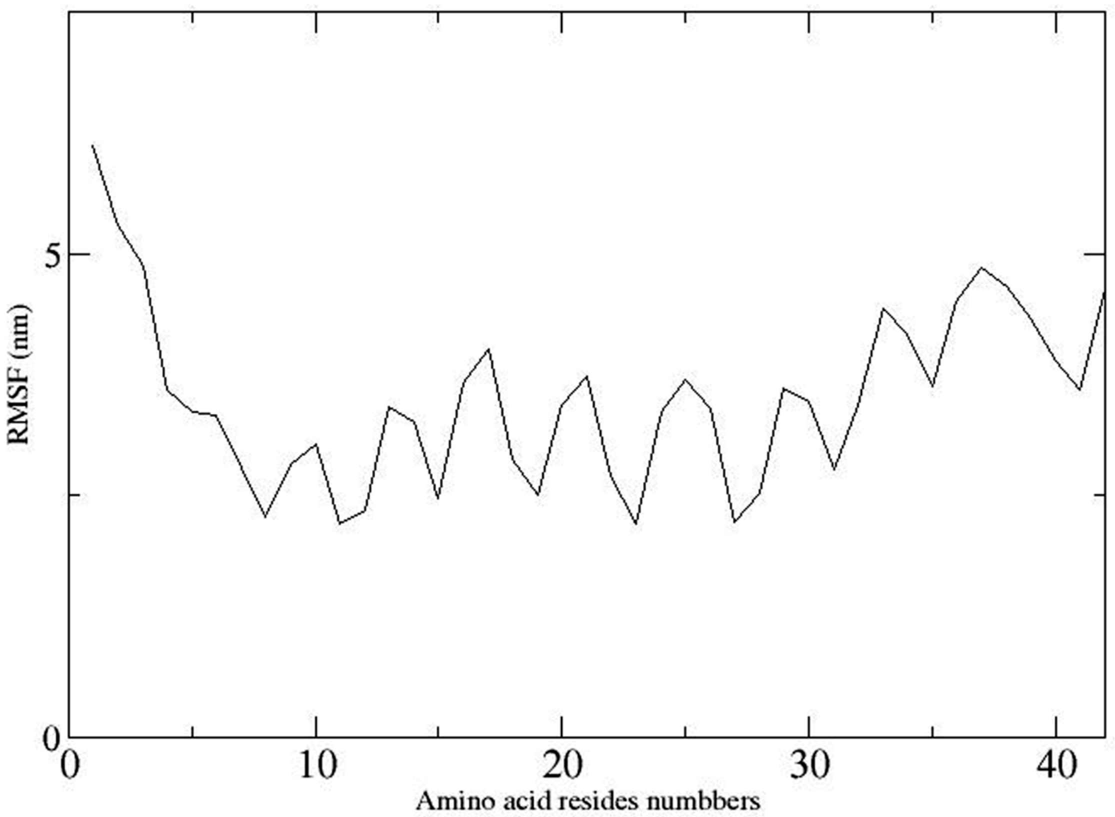

Figure S6. Root mean square fluctuation (RMSF) plot of protein residues of compound 2-AB42 MD simulations system.

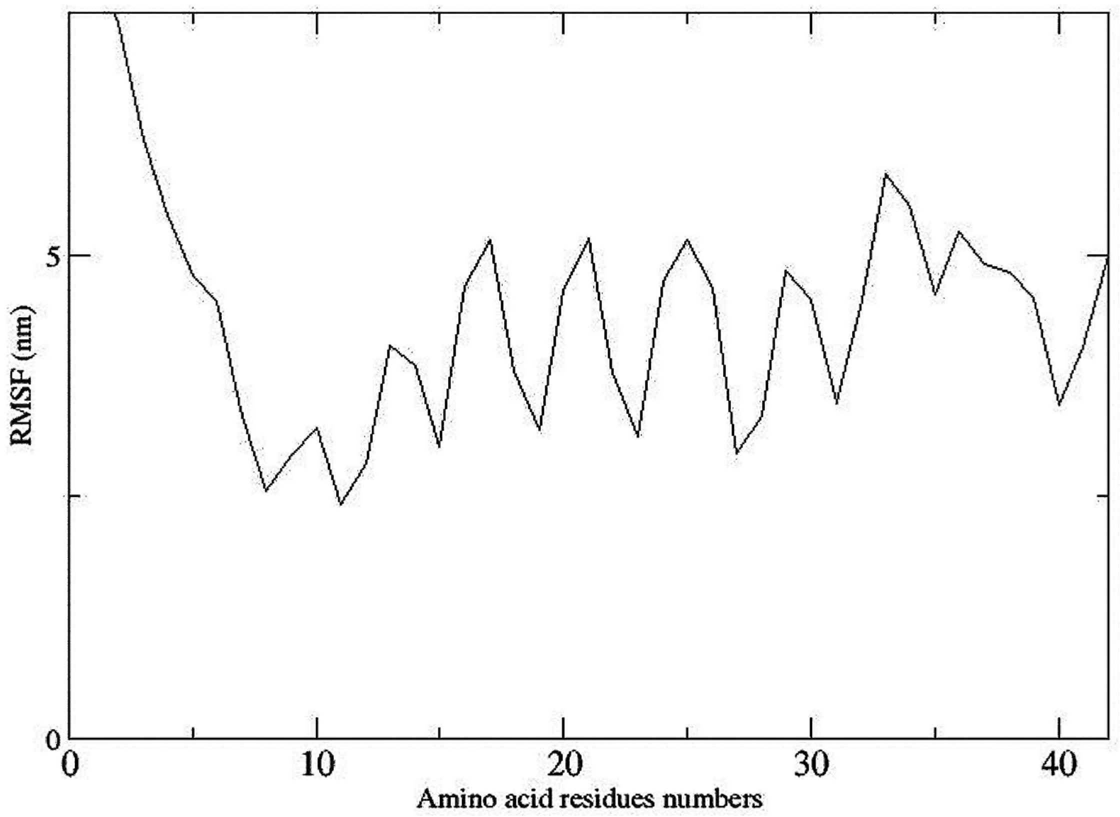

Figure S7. Root mean square fluctuation (RMSF) plot of protein residues of compound 3-AB42 MD simulations system.

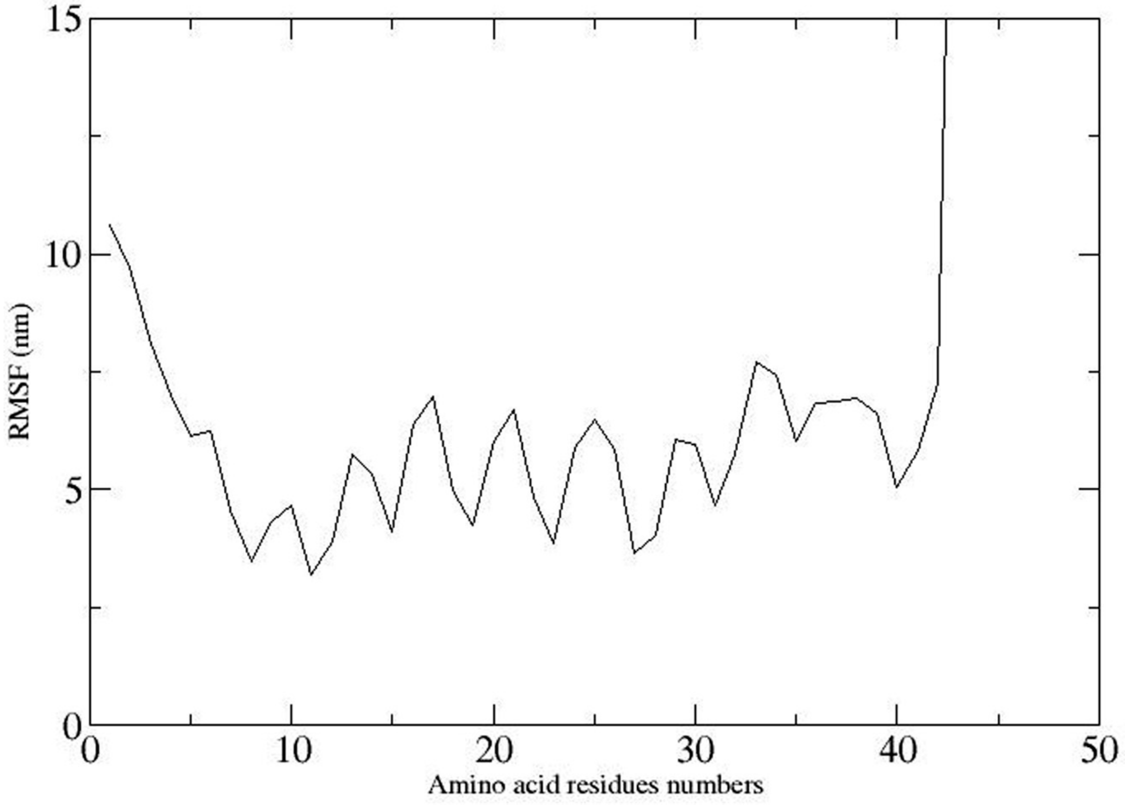

Figure S8. Root mean square fluctuation (RMSF) plot of protein residues of compound 4-AB42 MD simulations system.

Table S1. Hydrogen bonds between hydrogen donor atoms of compound 1 (UNK) and hydrogen acceptor atoms of aminoacid residues of AB42.

#Acceptor	DonorH	Donor	Frames	Frac	AvgDist	AvgAng
GLU_22@OE2	UNK_43@H2	UNK_43@O3	176	0.22	2.6493	164.3179
GLN_15@OE1	UNK_43@H2	UNK_43@O3	71	0.0112	2.7194	164.7692
GLU_22@OE1	UNK_43@H2	UNK_43@O3	33	0.0413	2.6349	163.2027
SER_26@OG	*UNK_43@H2*	*UNK_43@O3*	*19*	*0.0887*	*2.7675*	*164.0891*
GLN_15@OE1	UNK_43@H2	UNK_43@O3	9	0.0112	2.7194	164.7692
ASN_27@OD1	UNK_43@H2	UNK_43@O3	9	0.0112	2.7358	164.0929
TYR_10@OH	UNK_43@H	UNK_43@O1	9	0.0112	2.8376	160.4962
GLN_15@HE22	UNK_43@HC8	UNK_43@C15	9	0.0112	2.8857	151.1244
SER_26@HG	UNK_43@H2	UNK_43@O3	8	0.01	2.847	146.8737
GLU_22@O	UNK_43@H2	UNK_43@O3	4	0.005	2.7132	165.1355
ASN_27@HD21	UNK_43@HC10	UNK_43@C16	2	0.0025	2.7385	145.1437
GLN_15@HE22	UNK_43@HC13	UNK_43@C18	2	0.0025	2.8391	150.635
GLU_22@HB3	UNK_43@HC9	UNK_43@C15	2	0.0025	2.9382	137.3737
GLU_22@HG2	UNK_43@HC3	UNK_43@C8	2	0.0025	2.9818	143.2933
GLN_15@OE1	UNK_43@H1	UNK_43@O2	1	0.0013	2.7034	141.8668
LYS_28@HE3	UNK_43@HC16	UNK_43@C19	1	0.0013	2.781	143.0719
ASN_27@HD21	UNK_43@HC13	UNK_43@C18	1	0.0013	2.7857	154.6361
ASN_27@HD21	UNK_43@HC12	UNK_43@C18	1	0.0013	2.8546	152.1665
HIE_14@HE2	UNK_43@HC9	UNK_43@C15	1	0.0013	2.8623	136.1457
GLY_29@HA3	UNK_43@HC10	UNK_43@C16	1	0.0013	2.9177	139.6789
SER_26@HB2	UNK_43@HC	UNK_43@C	1	0.0013	2.9274	159.7953
ASP_23@HB2	UNK_43@HC12	UNK_43@C18	1	0.0013	2.9605	135.9208
GLN_15@HE22	UNK_43@HC10	UNK_43@C16	1	0.0013	2.9651	138.2303
GLU_22@HG2	UNK_43@HC4	UNK_43@C10	1	0.0013	2.9666	149.0877
HIE_14@HA	UNK_43@HC5	UNK_43@C11	1	0.0013	2.971	145.1526
PHE_19@HA	UNK_43@HC14	UNK_43@C19	1	0.0013	2.972	153.6014
VAL_18@HG11	UNK_43@HC11	UNK_43@C18	1	0.0013	2.9721	146.23
VAL_18@HG22	UNK_43@HC4	UNK_43@C10	1	0.0013	2.975	138.5094
GLU_22@HA	UNK_43@HC	UNK_43@C	1	0.0013	2.9787	149.2751
PHE_19@HA	UNK_43@HC9	UNK_43@C15	1	0.0013	2.9826	135.882
PHE_19@HA	UNK_43@HC11	UNK_43@C18	1	0.0013	2.983	135.304
GLY_29@HA3	UNK_43@HC16	UNK_43@C19	1	0.0013	2.9841	146.2609
ASN_27@HB2	UNK_43@HC8	UNK_43@C15	1	0.0013	2.9846	137.0074
VAL_18@HG12	UNK_43@HC1	UNK_43@C4	1	0.0013	2.9856	142.8582
GLU_22@HG3	UNK_43@HC7	UNK_43@C14	1	0.0013	2.9876	166.2095
ASN_27@HD22	UNK_43@HC13	UNK_43@C18	1	0.0013	2.9917	154.8208
GLU_22@HG3	UNK_43@HC3	UNK_43@C8	1	0.0013	2.9972	170.2719
GLN_15@HE22	UNK_43@HC9	UNK_43@C15	1	0.0013	2.9993	167.1286

Table S2. Hydrogen bonds between hydrogen acceptor atoms of compound 1 (UNK) and hydrogen donor atoms of aminoacid residues of AB42.

#Acceptor	DonorH	Donor	Frames	Frac	AvgDist	AvgAng
UNK_43@O	GLN_15@HE22	GLN_15@NE2	67	0.0838	2.8835	158.1107
UNK_43@O3	ASN_27@HD21	ASN_27@ND2	16	0.02	2.872	149.3433
UNK_43@O3	SER_26@H	SER_26@N	13	0.0163	2.9316	160.7993
UNK_43@O1	HIE_14@HE2	HIE_14@NE2	11	0.0138	2.9064	152.3428
UNK_43@O	GLN_15@HE22	GLN_15@NE2	11	0.0138	2.8835	158.1107
UNK_43@O2	GLN_15@HE22	GLN_15@NE2	8	0.01	2.8606	156.9088
UNK_43@O2	HIE_14@HE2	HIE_14@NE2	8	0.01	2.8902	154.5428
UNK_43@HC8	GLN_15@HE22	GLN_15@NE2	6	0.0075	2.7568	154.6674
UNK_43@O1	HIE_13@HE2	HIE_13@NE2	5	0.0063	2.9194	152.61
UNK_43@H2	GLU_22@HA	GLU_22@CA	5	0.0063	2.9534	165.0206
UNK_43@C12	HIE_14@HE2	HIE_14@NE2	4	0.005	2.9487	147.2067
UNK_43@O3	GLN_15@HE22	GLN_15@NE2	4	0.005	2.958	149.8044
UNK_43@HC10	ASN_27@HD21	ASN_27@ND2	3	0.0037	2.7023	145.5778
UNK_43@H	HIE_14@HE2	HIE_14@NE2	3	0.0037	2.8497	152.3441
UNK_43@H2	ASN_27@HD21	ASN_27@ND2	3	0.0037	2.9237	138.997
UNK_43@O	HIE_14@HE2	HIE_14@NE2	3	0.0037	2.9388	155.6875
UNK_43@H1	GLN_15@HE22	GLN_15@NE2	3	0.0037	2.9634	151.7718
UNK_43@HC13	ASN_27@HD21	ASN_27@ND2	2	0.0025	2.8349	158.833
UNK_43@HC6	HIE_14@HE2	HIE_14@NE2	2	0.0025	2.8556	145.5849
UNK_43@HC	SER_26@HB2	SER_26@CB	2	0.0025	2.9085	141.4221
UNK_43@HC9	GLN_15@HE22	GLN_15@NE2	2	0.0025	2.9313	141.9867
UNK_43@HC14	PHE_19@HA	PHE_19@CA	2	0.0025	2.9323	137.2233
UNK_43@HC11	PHE_19@HA	PHE_19@CA	2	0.0025	2.9358	136.363
UNK_43@H2	PHE_19@HA	PHE_19@CA	2	0.0025	2.9552	158.0104
UNK_43@HC9	GLU_22@HB3	GLU_22@CB	2	0.0025	2.9617	142.7781
UNK_43@HC4	GLU_22@HB2	GLU_22@CB	2	0.0025	2.9704	138.3055
UNK_43@HC	GLU_22@HA	GLU_22@CA	2	0.0025	2.9705	144.3828
UNK_43@HC13	GLN_15@HE22	GLN_15@NE2	1	0.0013	2.5174	143.5805
UNK_43@H2	PHE_19@HD2	PHE_19@CD2	1	0.0013	2.7704	138.6219
UNK_43@O1	TYR_10@HH	TYR_10@OH	1	0.0013	2.7723	151.6032
UNK_43@HC10	GLN_15@HE22	GLN_15@NE2	1	0.0013	2.7769	136.9353
UNK_43@HC13	ASN_27@HD22	ASN_27@ND2	1	0.0013	2.7864	136.8778
UNK_43@HC3	GLU_22@HG3	GLU_22@CG	1	0.0013	2.8147	136.8553
UNK_43@O	SER_26@H	SER_26@N	1	0.0013	2.8177	137.452
UNK_43@HC16	LYS_28@HE3	LYS_28@CE	1	0.0013	2.866	158.2539
UNK_43@HC2	HIE_14@HE2	HIE_14@NE2	1	0.0013	2.8867	146.1952
UNK_43@O3	ASN_27@HD22	ASN_27@ND2	1	0.0013	2.904	146.5965

Table S3. Hydrogen bonds between hydrogen donor atoms of compound 2 (UNK) and hydrogen acceptor atoms of aminoacid residues of AB42.

#Acceptor	DonorH	Donor	Frames	Frac	AvgDist	AvgAng
GLU_22@OE1	UNK_43@H1	UNK_43@O2	998	0.998	2.5769	164.749
VAL_18@HG12	UNK_43@HC17	UNK_43@C20	3	0.003	2.951	153.5261
LEU_17@HD12	UNK_43@HC10	UNK_43@C16	2	0.002	2.9087	139.9779
VAL_18@HG22	UNK_43@HC17	UNK_43@C20	2	0.002	2.9126	139.088
VAL_18@HG12	UNK_43@HC18	UNK_43@C20	1	0.001	2.816	141.3296
GLY_25@HA3	UNK_43@HC2	UNK_43@C7	1	0.001	2.8677	141.072
ALA_21@HB3	UNK_43@HC18	UNK_43@C20	1	0.001	2.8708	138.6625
LEU_17@HD21	UNK_43@HC13	UNK_43@C18	1	0.001	2.8986	144.018
VAL_18@HG21	UNK_43@HC10	UNK_43@C16	1	0.001	2.9105	137.256
LEU_17@HD21	UNK_43@HC17	UNK_43@C20	1	0.001	2.9199	138.389
GLU_22@HB2	UNK_43@HC	UNK_43@C1	1	0.001	2.9321	136.5975
ALA_21@HB3	UNK_43@HC17	UNK_43@C20	1	0.001	2.9359	137.8585
LEU_17@HD21	UNK_43@HC15	UNK_43@C19	1	0.001	2.9368	141.3855
ALA_21@HB1	UNK_43@HC18	UNK_43@C20	1	0.001	2.955	160.3705
ALA_21@HB2	UNK_43@HC14	UNK_43@C19	1	0.001	2.9612	137.2148
ALA_21@HB1	UNK_43@HC13	UNK_43@C18	1	0.001	2.9625	144.9056
GLU_22@HB2	UNK_43@HC18	UNK_43@C20	1	0.001	2.9739	148.9507
ALA_21@HB3	UNK_43@HC14	UNK_43@C19	1	0.001	2.9832	137.0394
LEU_17@HD13	UNK_43@HC16	UNK_43@C19	1	0.001	2.9846	144.2506
VAL_18@HA	UNK_43@HC18	UNK_43@C20	1	0.001	2.9851	144.288
LEU_17@HD12	UNK_43@HC8	UNK_43@C15	1	0.001	2.988	137.0284
SER_26@HG	UNK_43@H1	UNK_43@O2	1	0.001	2.9889	136.6402
GLU_22@CD	UNK_43@H1	UNK_43@O2	1	0.001	2.9906	147.8
LEU_17@HB3	UNK_43@HC10	UNK_43@C16	1	0.001	2.9962	148.4131
VAL_18@HA	UNK_43@HC17	UNK_43@C20	1	0.001	2.9972	136.5001
LEU_17@HD23	UNK_43@HC14	UNK_43@C19	1	0.001	2.9988	145.4996

Table S4. Hydrogen bonds between hydrogen acceptor atoms of compound 2 (UNK) and hydrogen acceptor atoms of aminoacid residues of AB42.

#Acceptor	DonorH	Donor	Frames	Frac	AvgDist	AvgAng
UNK_43@O2	SER_26@H	SER_26@N	169	0.169	2.9189	158.3143
UNK_43@H1	SER_26@HG	SER_26@OG	36	0.036	2.8895	147.1838
UNK_43@H1	GLU_22@HA	GLU_22@CA	25	0.025	2.9397	157.3678
UNK_43@H1	SER_26@H	SER_26@N	8	0.008	2.9381	158.7508
UNK_43@O2	SER_26@HG	SER_26@OG	6	0.006	2.8221	159.7397
UNK_43@HC6	GLY_29@HA3	GLY_29@CA	6	0.006	2.9604	149.9789
UNK_43@HC7	GLY_25@HA2	GLY_25@CA	3	0.003	2.909	142.969
UNK_43@HC17	VAL_18@HG22	VAL_18@CG2	3	0.003	2.9694	143.8779
UNK_43@HC	GLU_22@HA	GLU_22@CA	2	0.002	2.8809	137.1575

UNK_43@HC18	VAL_18@HG12	VAL_18@CG1	2	0.002	2.8971	147.2648
UNK_43@HC17	VAL_18@HG12	VAL_18@CG1	2	0.002	2.9281	149.1059
UNK_43@HC19	VAL_18@HG21	VAL_18@CG2	2	0.002	2.9592	143.662
UNK_43@HC4	SER_26@HB3	SER_26@CB	2	0.002	2.9759	146.1827
UNK_43@HC4	SER_26@HB2	SER_26@CB	2	0.002	2.9855	140.0855
UNK_43@HC5	GLY_29@HA3	GLY_29@CA	2	0.002	2.9863	146.175
UNK_43@HC10	ALA_21@HB1	ALA_21@CB	1	0.001	2.8499	135.8965
UNK_43@HC13	LEU_17@HD11	LEU_17@CD1	1	0.001	2.8855	135.6917
UNK_43@HC13	LEU_17@HD21	LEU_17@CD2	1	0.001	2.8867	143.2838
UNK_43@HC11	LEU_17@HD21	LEU_17@CD2	1	0.001	2.8878	137.9249
UNK_43@HC9	VAL_18@HG22	VAL_18@CG2	1	0.001	2.8904	136.0827
UNK_43@HC2	GLY_25@HA3	GLY_25@CA	1	0.001	2.9302	140.0827
UNK_43@HC14	LEU_17@HD13	LEU_17@CD1	1	0.001	2.9379	141.1589
UNK_43@HC14	PHE_20@HE2	PHE_20@CE2	1	0.001	2.9402	145.177
UNK_43@HC16	LEU_17@HD22	LEU_17@CD2	1	0.001	2.9436	142.0576
UNK_43@HC18	VAL_18@HG22	VAL_18@CG2	1	0.001	2.9508	142.4419
UNK_43@HC10	LEU_17@HD12	LEU_17@CD1	1	0.001	2.9555	135.5255
UNK_43@HC19	VAL_18@HG12	VAL_18@CG1	1	0.001	2.9711	142.8912
UNK_43@HC16	ALA_21@HB2	ALA_21@CB	1	0.001	2.9805	139.1919
UNK_43@HC11	LEU_17@HD23	LEU_17@CD2	1	0.001	2.9905	144.0687
UNK_43@HC18	VAL_18@HG21	VAL_18@CG2	1	0.001	2.9974	159.2368
UNK_43@HC17	VAL_18@HA	VAL_18@CA	1	0.001	2.9997	151.8563

Table S5. Hydrogen bonds between hydrogen donor atoms of compound 3 (UNK) and hydrogen acceptor atoms of aminoacid residues of AB42.

#Acceptor	DonorH	Donor	Frames	Frac	AvgDist	AvgAng
ASP_7@OD1	UNK_43@H	UNK_43@O2	273	0.273	2.6845	163.5782
GLU_11@OE2	UNK_43@H	UNK_43@O2	135	0.135	2.6653	163.1702
ASP_7@OD2	UNK_43@H	UNK_43@O2	134	0.134	2.655	164.4232
GLU_11@OE1	UNK_43@H	UNK_43@O2	114	0.114	2.6563	164.063
ASN_27@HD21	UNK_43@HC10	UNK_43@C16	8	0.008	2.9022	149.0629
ASN_27@HD21	UNK_43@HC1	UNK_43@C8	6	0.006	2.8594	142.3632
ASP_23@HB2	UNK_43@HC11	UNK_43@C17	4	0.004	2.9394	140.4972
HIE_14@ND1	UNK_43@H	UNK_43@O2	3	0.003	2.8078	170.5725
ASP_7@O	UNK_43@H	UNK_43@O2	3	0.003	2.8143	161.5649
GLN_15@OE1	UNK_43@H	UNK_43@O2	2	0.002	2.6838	170.3025
TYR_10@HD1	UNK_43@HC7	UNK_43@C14	2	0.002	2.881	136.6021
ASP_7@CG	UNK_43@H	UNK_43@O2	2	0.002	2.9082	136.2841
PHE_19@HA	UNK_43@HC3	UNK_43@C2	2	0.002	2.937	141.4654
ASN_27@HD21	UNK_43@HC2	UNK_43@C2	2	0.002	2.9487	146.6546
PHE_19@HA	UNK_43@HC11	UNK_43@C17	2	0.002	2.9585	140.2634
GLU_22@HB2	UNK_43@HC9	UNK_43@C16	2	0.002	2.9634	145.0556
VAL_18@HG13	UNK_43@HC8	UNK_43@C15	2	0.002	2.9678	145.1266
TYR_10@HE1	UNK_43@HC7	UNK_43@C14	2	0.002	2.9741	149.1246

GLU_22@HB2	UNK_43@HC11	UNK_43@C17	2	0.002	2.9881	137.9211
ASN_27@HD21	UNK_43@H2	UNK_43@C1	1	0.001	2.8161	138.762
VAL_18@HB	UNK_43@HC8	UNK_43@C15	1	0.001	2.8581	139.4856
GLU_22@HG3	UNK_43@H2	UNK_43@C1	1	0.001	2.8711	135.2135
GLN_15@HE21	UNK_43@HC3	UNK_43@C2	1	0.001	2.8735	139.1597
PHE_19@HA	UNK_43@HC1	UNK_43@C8	1	0.001	2.8796	136.4306
GLN_15@HA	UNK_43@HC2	UNK_43@C2	1	0.001	2.9221	140.1796
ASN_27@HA	UNK_43@HC15	UNK_43@C20	1	0.001	2.9408	150.1402
GLU_22@HG3	UNK_43@HC7	UNK_43@C14	1	0.001	2.9428	137.9243
HIE_14@HB3	UNK_43@HC7	UNK_43@C14	1	0.001	2.9436	137.5188
GLN_15@HG2	UNK_43@HC3	UNK_43@C2	1	0.001	2.9442	137.6795
HIE_14@HD2	UNK_43@HC6	UNK_43@C12	1	0.001	2.9512	165.3662
PHE_19@HD2	UNK_43@HC1	UNK_43@C8	1	0.001	2.9523	140.4736
VAL_18@HG12	UNK_43@HC5	UNK_43@C11	1	0.001	2.9568	138.713
ASP_23@HA	UNK_43@HC12	UNK_43@C19	1	0.001	2.9606	138.6758
VAL_18@HG11	UNK_43@HC8	UNK_43@C15	1	0.001	2.9607	136.157
GLU_22@HG3	UNK_43@H11	UNK_43@C1	1	0.001	2.9642	144.0151
SER_26@HB3	UNK_43@HC10	UNK_43@C16	1	0.001	2.9728	150.8447
VAL_18@HG11	UNK_43@HC5	UNK_43@C11	1	0.001	2.9737	137.7317
GLU_11@CD	UNK_43@H	UNK_43@O2	1	0.001	2.9737	136.7977
ASP_23@HB2	UNK_43@HC17	UNK_43@C20	1	0.001	2.9766	135.4272
GLU_22@HG3	UNK_43@HC3	UNK_43@C2	1	0.001	2.978	135.687
ASP_23@HB2	UNK_43@HC16	UNK_43@C20	1	0.001	2.9807	142.6022
PHE_19@HA	UNK_43@HC16	UNK_43@C20	1	0.001	2.9861	138.9571
GLU_22@HG3	UNK_43@HC5	UNK_43@C11	1	0.001	2.9875	139.2975
GLU_11@HG3	UNK_43@HC7	UNK_43@C14	1	0.001	2.9901	147.3732
GLU_22@HG3	UNK_43@H12	UNK_43@C1	1	0.001	2.9904	135.1153
GLU_11@HG3	UNK_43@HC6	UNK_43@C12	1	0.001	2.998	139.3394

Table S6. Hydrogen bonds between hydrogen donor atoms of compound 3 (UNK) and hydrogen acceptor atoms of aminoacid residues of AB42.

#Acceptor	DonorH	Donor	Frames	Frac	AvgDist	AvgAng
UNK_43@O	ASN_27@HD21	ASN_27@ND2	113	0.113	2.8621	159.0153
UNK_43@HC10	ASN_27@HD21	ASN_27@ND2	15	0.015	2.8405	150.9224
UNK_43@O3	ASN_27@HD21	ASN_27@ND2	10	0.01	2.8953	152.2543
UNK_43@HC2	GLN_15@HA	GLN_15@CA	7	0.007	2.9806	137.8808
UNK_43@O	GLN_15@HE21	GLN_15@NE2	6	0.006	2.8071	154.0959
UNK_43@HC1	ASN_27@HD21	ASN_27@ND2	6	0.006	2.8092	142.1934
UNK_43@H12	ASN_27@HD21	ASN_27@ND2	5	0.005	2.7252	137.4573
UNK_43@HC14	ASN_27@HD21	ASN_27@ND2	5	0.005	2.8163	143.4109
UNK_43@HC13	ASN_27@HD21	ASN_27@ND2	5	0.005	2.8461	155.8723
UNK_43@H2	ASN_27@HD21	ASN_27@ND2	5	0.005	2.873	143.3265

UNK_43@HC9	ASN_27@HD21	ASN_27@ND2	4	0.004	2.9189	148.986
UNK_43@H12	GLU_22@HG3	GLU_22@CG	4	0.004	2.9258	139.9264
UNK_43@HC11	PHE_19@HA	PHE_19@CA	4	0.004	2.9614	138.591
UNK_43@HC1	PHE_19@HA	PHE_19@CA	4	0.004	2.9706	141.0069
UNK_43@HC6	HIE_14@HD2	HIE_14@CD2	3	0.003	2.8896	141.2978
UNK_43@HC12	ASN_27@HD21	ASN_27@ND2	3	0.003	2.9043	152.3763
UNK_43@HC11	ASP_23@HB2	ASP_23@CB	3	0.003	2.9469	141.4237
UNK_43@O2	GLN_15@HE22	GLN_15@NE2	3	0.003	2.9605	161.2087
UNK_43@C17	ASN_27@HD21	ASN_27@ND2	3	0.003	2.9837	153.7535
UNK_43@HC8	VAL_18@HB	VAL_18@CB	2	0.002	2.8846	142.6994
UNK_43@H11	ASN_27@HD21	ASN_27@ND2	2	0.002	2.887	141.8896
UNK_43@HC10	GLU_22@HB3	GLU_22@CB	2	0.002	2.899	137.522
UNK_43@H	GLU_11@HG3	GLU_11@CG	2	0.002	2.9261	144.4856
UNK_43@HC1	PHE_19@HD2	PHE_19@CD2	2	0.002	2.9448	143.2694
UNK_43@HC7	GLU_11@HG3	GLU_11@CG	2	0.002	2.9594	137.5654
UNK_43@HC3	PHE_19@HA	PHE_19@CA	2	0.002	2.9682	146.241
UNK_43@HC3	GLU_22@HG3	GLU_22@CG	2	0.002	2.9749	139.4038
UNK_43@HC15	ASN_27@HA	ASN_27@CA	2	0.002	2.9777	138.6418
UNK_43@H	HIE_14@HD2	HIE_14@CD2	1	0.001	2.7616	138.5507
UNK_43@HC17	ASN_27@HD21	ASN_27@ND2	1	0.001	2.7742	143.2859
UNK_43@HC7	HIE_14@HE2	HIE_14@NE2	1	0.001	2.8295	139.4341
UNK_43@H2	ILE_31@HD13	ILE_31@CD1	1	0.001	2.8476	141.3626
UNK_43@HC3	GLN_15@HE21	GLN_15@NE2	1	0.001	2.8764	135.4483
UNK_43@HC11	ASN_27@HD21	ASN_27@ND2	1	0.001	2.8928	135.0968
UNK_43@HC7	TYR_10@HE1	TYR_10@CE1	1	0.001	2.8978	147.6636
UNK_43@C10	ASN_27@HD21	ASN_27@ND2	1	0.001	2.9171	136.121
UNK_43@H11	GLU_22@HG3	GLU_22@CG	1	0.001	2.9189	141.7294
UNK_43@H11	SER_26@HG	SER_26@OG	1	0.001	2.9205	152.408
UNK_43@HC3	GLN_15@HG2	GLN_15@CG	1	0.001	2.9226	135.9704
UNK_43@HC10	GLU_22@HB2	GLU_22@CB	1	0.001	2.9246	135.1701
UNK_43@HC	PHE_19@HD2	PHE_19@CD2	1	0.001	2.9281	137.6789
UNK_43@HC17	ALA_30@HB3	ALA_30@CB	1	0.001	2.9323	141.628
UNK_43@HC10	ALA_30@HB2	ALA_30@CB	1	0.001	2.9332	145.8013
UNK_43@HC7	GLU_22@HG3	GLU_22@CG	1	0.001	2.9431	137.1692
UNK_43@HC8	PHE_19@HA	PHE_19@CA	1	0.001	2.9469	139.704
UNK_43@H11	GLU_22@HB3	GLU_22@CB	1	0.001	2.9546	143.6952
UNK_43@HC16	ASP_23@HB2	ASP_23@CB	1	0.001	2.9579	140.5616
UNK_43@O2	HIE_14@HE2	HIE_14@NE2	1	0.001	2.9654	135.4561
UNK_43@HC16	PHE_19@HA	PHE_19@CA	1	0.001	2.9664	137.5227
UNK_43@HC15	PHE_19@HA	PHE_19@CA	1	0.001	2.9687	138.0649
UNK_43@HC7	HIE_14@HD2	HIE_14@CD2	1	0.001	2.9818	153.3283
UNK_43@O1	ASN_27@HD21	ASN_27@ND2	1	0.001	2.9828	136.3547
UNK_43@HC7	VAL_18@HG11	VAL_18@CG1	1	0.001	2.9834	144.0709
UNK_43@HC11	ALA_30@HB3	ALA_30@CB	1	0.001	2.9849	140.3635
UNK_43@HC9	ALA_30@HB2	ALA_30@CB	1	0.001	2.9909	142.0212

UNK_43@HC3	GLN_15@HG3	GLN_15@CG	1	0.001	2.9932	136.8299
UNK_43@HC8	GLN_15@HE21	GLN_15@NE2	1	0.001	2.994	156.4887
UNK_43@HC2	GLU_22@HG3	GLU_22@CG	1	0.001	2.9951	135.3212
UNK_43@H2	GLU_22@HG3	GLU_22@CG	1	0.001	2.9969	145.129
UNK_43@HC7	HIE_14@HB3	HIE_14@CB	1	0.001	2.9996	149.8881

Table S7. Hydrogen bonds between hydrogen donor atoms of compound 4 (UNK) and hydrogen acceptor atoms of aminoacid residues of AB42.

#Acceptor	DonorH	Donor	Frames	Frac	AvgDist	AvgAng
GLY_33@O	UNK_43@H1	UNK_43@O3	5	0.005	2.7673	162.5818
SER_26@HA	UNK_43@HC2	UNK_43@C1	4	0.004	2.9177	138.0418
GLY_29@HA3	UNK_43@HC5	UNK_43@C10	4	0.004	2.9522	146.4538
GLU_22@OE2	UNK_43@H	UNK_43@O2	3	0.003	2.7269	158.9864
GLY_29@HA3	UNK_43@HC2	UNK_43@C1	3	0.003	2.9452	137.8545
ALA_30@O	UNK_43@H1	UNK_43@O3	2	0.002	2.7714	144.991
GLY_29@HA3	UNK_43@HC8	UNK_43@C14	2	0.002	2.8741	138.5713
LEU_34@HB2	UNK_43@HC11	UNK_43@C16	2	0.002	2.952	141.6181
ALA_30@HB2	UNK_43@HC13	UNK_43@C18	2	0.002	2.9557	138.1869
LEU_34@HB2	UNK_43@HC17	UNK_43@C19	2	0.002	2.986	143.973
SER_26@OG	UNK_43@H	UNK_43@O2	1	0.001	2.8293	164.0437
ASN_27@HA	UNK_43@HC17	UNK_43@C19	1	0.001	2.8666	135.022
LEU_34@HD11	UNK_43@HC11	UNK_43@C16	1	0.001	2.8814	135.1464
ALA_30@HA	UNK_43@HC2	UNK_43@C1	1	0.001	2.8904	135.9276
SER_26@HB2	UNK_43@HC15	UNK_43@C19	1	0.001	2.9153	141.1875
LEU_34@HD23	UNK_43@HC15	UNK_43@C19	1	0.001	2.9215	138.954
GLY_29@HA3	UNK_43@HC4	UNK_43@C8	1	0.001	2.9313	138.2309
GLY_29@O	UNK_43@H1	UNK_43@O3	1	0.001	2.9444	157.3882
LEU_34@HB2	UNK_43@HC12	UNK_43@C18	1	0.001	2.9496	139.0069
SER_26@HG	UNK_43@HC2	UNK_43@C1	1	0.001	2.9543	146.3262
GLY_33@HA3	UNK_43@HC9	UNK_43@C15	1	0.001	2.9554	139.0671
GLY_33@HA3	UNK_43@HC11	UNK_43@C16	1	0.001	2.9578	149.0206
GLY_33@HA3	UNK_43@HC14	UNK_43@C18	1	0.001	2.9614	138.3979
SER_26@HG	UNK_43@HC16	UNK_43@C19	1	0.001	2.9665	156.4123
LEU_34@HD23	UNK_43@HC9	UNK_43@C15	1	0.001	2.9686	138.7635
LEU_34@HB2	UNK_43@HC13	UNK_43@C18	1	0.001	2.9709	140.556
GLY_33@HA2	UNK_43@HC10	UNK_43@C15	1	0.001	2.9771	136.6127
GLY_33@HA3	UNK_43@HC4	UNK_43@C8	1	0.001	2.9773	137.8045
ILE_32@HG23	UNK_43@HC7	UNK_43@C13	1	0.001	2.9849	135.9945
GLY_29@HA3	UNK_43@HC6	UNK_43@C11	1	0.001	2.9856	139.4816
ILE_32@HG21	UNK_43@HC6	UNK_43@C11	1	0.001	2.9857	141.0134
LEU_34@HG	UNK_43@HC10	UNK_43@C15	1	0.001	2.9866	136.5684

ALA_30@HB2	UNK_43@HC9	UNK_43@C15	1	0.001	2.9919	136.3156
SER_26@HB3	UNK_43@HC3	UNK_43@C1	1	0.001	2.9927	136.111
ALA_30@HB2	UNK_43@HC2	UNK_43@C1	1	0.001	2.9933	137.4706

Table S8. Hydrogen bonds between hydrogen acceptor atoms of compound 4 (UNK) and hydrogen acceptor atoms of aminoacid residues of AB42.

#Acceptor	DonorH	Donor	Frames	Frac	AvgDist	AvgAng
ALA_30@O	UNK_43@H1	UNK_43@O3	6	0.003	2.716	156.2652
GLY_33@O	UNK_43@H1	UNK_43@O3	5	0.0025	2.7673	162.5818
SER_26@HA	UNK_43@HC2	UNK_43@C1	4	0.002	2.9177	138.0418
GLY_29@HA3	UNK_43@HC5	UNK_43@C10	4	0.002	2.9522	146.4538
GLU_22@OE2	UNK_43@H	UNK_43@O2	3	0.0015	2.7269	158.9864
GLY_29@HA3	UNK_43@HC2	UNK_43@C1	3	0.0015	2.9452	137.8545
GLY_33@HA3	UNK_43@HC	UNK_43@C	3	0.0015	2.9827	137.9944
GLY_25@O	UNK_43@H	UNK_43@O2	2	0.001	2.6084	169.8516
GLY_29@HA3	UNK_43@HC8	UNK_43@C14	2	0.001	2.8741	138.5713
GLY_29@HA3	UNK_43@HC	UNK_43@C	2	0.001	2.9265	143.772
LEU_34@HB2	UNK_43@HC11	UNK_43@C16	2	0.001	2.952	141.6181
ALA_30@HB2	UNK_43@HC13	UNK_43@C18	2	0.001	2.9557	138.1869
SER_26@HB2	UNK_43@HC7	UNK_43@C13	2	0.001	2.9694	137.3632
LEU_34@HB2	UNK_43@HC17	UNK_43@C19	2	0.001	2.986	143.973
SER_26@OG	UNK_43@H	UNK_43@O2	1	0.0005	2.8293	164.0437
ASN_27@HA	UNK_43@HC17	UNK_43@C19	1	0.0005	2.8666	135.022
LEU_34@HB2	UNK_43@HC15	UNK_43@C19	1	0.0005	2.8678	137.5228
SER_26@HB2	UNK_43@HC4	UNK_43@C8	1	0.0005	2.8791	137.6025
LEU_34@HD11	UNK_43@HC11	UNK_43@C16	1	0.0005	2.8814	135.1464
ALA_30@HA	UNK_43@HC2	UNK_43@C1	1	0.0005	2.8904	135.9276
GLY_33@HA3	UNK_43@HC10	UNK_43@C15	1	0.0005	2.9016	145.8792
SER_26@HB2	UNK_43@HC15	UNK_43@C19	1	0.0005	2.9153	141.1875
LEU_34@HD23	UNK_43@HC15	UNK_43@C19	1	0.0005	2.9215	138.954
ARG_5@HD2	UNK_43@HC5	UNK_43@C10	1	0.0005	2.9279	145.0713
GLY_29@HA3	UNK_43@HC4	UNK_43@C8	1	0.0005	2.9313	138.2309
VAL_36@HG23	UNK_43@HC11	UNK_43@C16	1	0.0005	2.9325	138.2447
GLY_29@O	UNK_43@H1	UNK_43@O3	1	0.0005	2.9444	157.3882
LEU_34@HB2	UNK_43@HC12	UNK_43@C18	1	0.0005	2.9496	139.0069
SER_26@HG	UNK_43@HC2	UNK_43@C1	1	0.0005	2.9543	146.3262
GLY_33@HA3	UNK_43@HC9	UNK_43@C15	1	0.0005	2.9554	139.0671
GLY_33@HA3	UNK_43@HC11	UNK_43@C16	1	0.0005	2.9578	149.0206
GLY_29@HA2	UNK_43@HC5	UNK_43@C10	1	0.0005	2.9595	144.9722
GLY_33@HA3	UNK_43@HC14	UNK_43@C18	1	0.0005	2.9614	138.3979
ALA_30@HA	UNK_43@HC12	UNK_43@C18	1	0.0005	2.9652	138.6842

SER_26@HG	UNK_43@HC16	UNK_43@C19	1	0.0005	2.9665	156.4123
LEU_34@HD23	UNK_43@HC9	UNK_43@C15	1	0.0005	2.9686	138.7635
GLY_33@HA3	UNK_43@HC13	UNK_43@C18	1	0.0005	2.9708	140.9673
LEU_34@HB2	UNK_43@HC13	UNK_43@C18	1	0.0005	2.9709	140.556
GLY_33@HA2	UNK_43@HC10	UNK_43@C15	1	0.0005	2.9771	136.6127
GLY_33@HA3	UNK_43@HC4	UNK_43@C8	1	0.0005	2.9773	137.8045
ILE_32@HG23	UNK_43@HC7	UNK_43@C13	1	0.0005	2.9849	135.9945
GLY_29@HA3	UNK_43@HC6	UNK_43@C11	1	0.0005	2.9856	139.4816
ILE_32@HG21	UNK_43@HC6	UNK_43@C11	1	0.0005	2.9857	141.0134
LEU_34@HG	UNK_43@HC10	UNK_43@C15	1	0.0005	2.9866	136.5684
ALA_30@HB2	UNK_43@HC9	UNK_43@C15	1	0.0005	2.9919	136.3156
SER_26@HB3	UNK_43@HC3	UNK_43@C1	1	0.0005	2.9927	136.111
ALA_30@HB2	UNK_43@HC2	UNK_43@C1	1	0.0005	2.9933	137.4706
LEU_34@HA	UNK_43@HC9	UNK_43@C15	1	0.0005	2.994	135.3266
GLY_25@HA2	UNK_43@HC6	UNK_43@C11	1	0.0005	2.9998	144.6449
